# Association between PaO_2_/(FiO_2_*PEEP) ratio and in-hospital mortality in COVID-19 patients: A reanalysis of published data from Peru using PaO_2_/(FiO_2_*PEEP) ratio in place of PaO_2_/FaO_2_ ratio

**DOI:** 10.1097/MD.0000000000039931

**Published:** 2024-10-04

**Authors:** Youli Chen, Huangen Li, Jinhuang Lin, Zhiwei Su, Tianlai Lin

**Affiliations:** a Intensive Care Unit, Fujian Medical University Affiliated First Quanzhou Hospital, Quanzhou, Fujian, PR China.

**Keywords:** COVID-19, mechanical ventilation, mortality, PaO_2_/FiO_2_, PEEP

## Abstract

P/FP [PaO_2_/(FiO_2_*PEEP)] is associated with in-hospital mortality in patients with acute respiratory distress syndrome (ARDS). However, to the best of our knowledge, the association between P/FP after 24 hours of invasive mechanical ventilation (IMV) and in-hospital mortality in patients with ARDS due to Coronavirus Disease 2019 (COVID-19) remained unclear. This study aimed to evaluate the relationship between the P/FP after 24 hours of IMV and in-hospital mortality in patients with ARDS due to COVID-19. We reanalyzed previously published data from Peru. Hueda-Zavaleta et al conducted a retrospective cohort study between April 2020 and April 2021 in southern Peru. A total of 200 hospitalized COVID-19 patients requiring IMV were included in this analysis. We used Cox proportional hazard regression models and Kaplan–Meier survival analysis to investigate the effect of P/FP after 24 hours of IMV on in-hospital mortality. We used a restricted cubic spline regression and a two-piecewise Cox proportional hazards model to explore the relationship between P/FP after 24 hours of IMV and in-hospital mortality in patients with ARDS due to COVID-19. Of the 200 patients, 51 (25.50%) died in hospital. The median P/FP was 20.45 mm Hg/cmH_2_O [interquartile range 15.79–25.21 mm Hg/cmH_2_O], with a range of 5.67 mm Hg/cmH_2_O to 51.21 mm Hg/cmH_2_O. Based on the P/FP ratio, patients were equally divided into 2 groups (low group [P/FP < 20.50 mm Hg/cmH_2_O] and high group [P/FP ≥ 20.50 mm Hg/cmH_2_O]). In-hospital mortality was lower in the high P/FP group than in the low P/FP group (12 [12%] vs 39 [39%]; unadjusted hazard ratio [HR]: 0.33, 95% confidence interval [CI]: 0.17–0.63; adjusted HR: 0.10, 95% CI: 0.02–0.47). We also found a nonlinear relationship between P/FP and in-hospital mortality. After adjusting for potential confounders, the HR was 0.67 (95% CI: 0.56–0.79) for P/FP ≤ 22 mm Hg/cmH_2_O and 1.10 (95% CI: 0.83–1.47) for P/FP > 22 mm Hg/cmH_2_O. In addition, lymphocytes ≤ 1 × 10^9^/L and acute kidney failure had a higher risk of death. After adjusting for potential confounders, the P/FP after 24 hours of IMV was nonlinearly associated with in-hospital mortality in patients with ARDS due to COVID-19.

## 1. Introduction

Arterial pressure of oxygen/inspiratory fraction of oxygen (PaO_2_/FiO_2_) is an important indicator for classifying the severity of acute respiratory distress syndrome (ARDS).^[1,2]^ ARDS caused by Coronavirus Disease 2019 (COVID-19) is the common cause of patient death.^[3,4]^ Most patients with ARDS due to COVID-19 require invasive mechanical ventilation (IMV).^[5]^ Hueda-Zavaleta et al^[6]^ found that PaO_2_/FiO_2_ after 24 hours of IMV was associated with in-hospital mortality in COVID-19 patients. The value of PaO_2_ was influenced by end-expiratory pressure (PEEP).^[7]^ Palanidurai S et al^[7]^ found that the P/FP [PaO_2_/(FiO_2_*PEEP)] was associated with mortality in patients with ARDS. However, to the best of our knowledge, the association between P/FP after 24 hours of IMV and in-hospital mortality in patients with ARDS due to COVID-19 remained unclear. In this study, we aimed to evaluate the relationship between P/FP after 24 hours of IMV and in-hospital mortality in patients with ARDS due to COVID-19.

## 2. Methods

### 2.1. Study design and participants

We reanalyzed previously published data from Peru. The retrospective cohort study was conducted by Hueda-Zavaleta et al^[6]^ between April 2020 and April 2021 in southern Peru. We reanalyzed data from 200 patients who were diagnosed with ARDS due to COVID-19 and required IMV treatment. Hueda-Zavaleta et al^[6]^ had previously described Clinical management strategies such as mechanical ventilation and prone positioning. The study protocol (N391-2021-UPT/FACSA-D) was approved by the ethics committee of the Faculty of Health Sciences of the Private University of Tacna. As this was a retrospective study, the informed consent was waived. The data were obtained freely from Hueda-Zavaleta et al^[6]^ (https://www.ncbi.nlm.nih.gov/pmc/articles/PMC9756861/bin/peerj-10-14290-s004.xls).

### 2.2. Stratification definitions

High white blood cells group: white blood cell count > 10 × 10^9^/L. High lymphocytes group: lymphocyte count > 1 × 10^9^/L. High procalcitonin group: procalcitonin concentration > 0.5 ng/mL. High C-reactive protein group: C-reactive protein level > 100 mg/L. High alanine aminotransferase group: alanine aminotransferase activity > 40 U/L. High aspartate aminotransferase group: aspartate aminotransferase activity > 40 U/L. High creatinine phosphokinase-total group: total creatine phosphokinase level > 200 U/L. High creatinine phosphokinase-MB group: creatine phosphokinase-MB isoform level > 25 U/L.^[8]^

High sequential organ failure assessment score group: sequential organ failure assessment score > 4.^[9]^

High plateau pressure after 24 hours of IMV group: plateau pressure > 30 cmH_2_O. High driving pressure after 24 hours of IMV group: driving pressure > 15 cmH_2_O.^[6]^

Lung damage on computed tomography: based on the percentage of lung involvement on computed tomography images, lung injuries were divided equally into 2 groups (low group [≤50%] and high group [>50%]).

### 2.3. Statistical analysis

Descriptive statistics were shown as median [interquartile range (IQR)] or number. Based on the P/FP ratio, patients were equally divided into 2 groups (low group [P/FP < 20.50 mm Hg/cmH_2_O] and high group [P/FP ≥ 20.50 mm Hg/cmH_2_O]). We used the Kruskal–Wallis test for continuous variables and the chi-square test or Fisher exact for categorical variables. Univariate and multivariate analyses were performed with a Cox proportional hazards model. To reduce missing data biases and maximize statistical power, we employed multiple imputation (MI). We used the stratified Cox proportional hazards model to compare in-hospital mortality in different groups. Interactions between subgroups were examined by likelihood ratio tests. We assessed the effect of P/FP on in-hospital mortality by constructing Kaplan–Meier curves and performing a log-rank test. To identify a nonlinear relationship, we applied a smooth curve technique to estimate the shape between P/FP and in-hospital mortality by restricted cubic spline regression. A two-piecewise Cox proportional hazards model was further performed to calculate the threshold effect of P/FP on in-hospital mortality using a smoothing plot. The inflection point was determined using the recursive method, where the maximum model likelihood was used. Furthermore, we performed a log-likelihood ratio test and compared the one-line linear regression model with the two-piece-wise linear model. All analyses were completed with EmpowerStats (www.empowerstats.com, X&Y solutions, Boston, MA) and R software version 3.6.1 (http://www.r-project.org). Statistical significance was defined when a two-tailed *P*-value was <.05.

## 3. Results

### 3.1. Patient and baseline characteristics

A total of 200 COVID-19 patients requiring IMV were included in this analysis. The demographic and clinical characteristics of patients were shown in Table 1 according to P/FP. There were statistical differences in comorbidities between the 2 groups, with hypertension, chronic kidney disease, and heart failure being more common in the low P/FP group. Furthermore, the low P/FP group showed higher C-reactive protein levels, more severe lung damage on computed tomography, a higher sequential organ failure assessment score, and elevated platform pressure levels. Additionally, sepsis, septic shock, acute kidney failure, pneumonia associated with IMV, colchicine administration, and renal replacement therapy were more common in the low P/FP group. The duration of IMV was longer in the low P/FP group compared with the high P/FP group (11 days [IQR: 8–19] vs 7 days [IQR: 4–13]). The duration of intensive care unit (ICU) was longer in the low P/FP group compared with the high P/FP group (12 days [IQR: 8–18] vs 8 days [IQR: 4–12.25]). Mortality was higher in the low P/FP group compared with the high P/FP group (39 [39%] vs 12 [12%]).

**Table 1:** Baseline characteristics by PaO_2_/(FiO_2_*PEEP).

Variable	Low (P/FP < 20.50)	High (P/FP ≥ 20.50)	*P*-value
Number	100	100	
Gender (female/male)	18/82	24/76	.298
Age (<65 years/≥65 years)	77/23	82/18	.381
Obesity (no/yes)	38/62	44/56	.388
Hypertension (no/yes)	67/33	80/20	.037
Diabetes (no/yes)	77/23	80/20	.606
Chronic kidney disease (no/yes)	92/8	100/0	.007
Heart failure (no/yes)	90/10	100/0	.001
Asthma (no/yes)	83/17	87/13	.428
Immunosuppression (no/yes)	88/12	93/6	.144
White blood cells (≤10 × 10^9^/L/>10 × 10^9^/L)	51/49	61/39	.154
Lymphocytes (≤1 × 10^9^/L/>1 × 10^9^/L)	75/25	72/28	.631
C-reactive protein (≤100 mg/L/>100 mg/L)	27/70	43/50	.009
Procalcitonin (≤0.5 ng/mL/>0.5 ng/mL)	57/19	53/7	.050
Alanine aminotransferase (≤40 U/L/>40 U/L)	30/70	25/75	.428
Aspartate aminotransferase (≤40 U/L/>40 U/L)	32/68	39/61	.301
Creatinine phosphokinase-total (≤200 U/L/>200 U/L)	62/31	60/29	.914
Creatinine phosphokinase-MB (≤25 U/L/>25 U/L)	41/55	49/43	.148
Lung damage on computed tomography (≤50%/>50%)	24/76	51/49	<.001
Sequential organ failure assessment (≤4/>4)	54/45	78/22	<.001
Colchicine (no/yes)	64/36	84/16	.001
Tocilizumab (no/yes)	84/16	90/10	.207
Renal replacement therapy (no/yes)	87/13	98/2	.003
Sepsis (no/yes)	19/81	38/62	.003
Septic shock (no/yes)	59/41	83/17	<.001
Acute kidney failure (no/yes)	74/26	97/3	<.001
Arrhythmia (no/yes)	92/8	94/5	.40
Pneumonia associated with IMV (no/yes)	71/29	85/15	.017
Catheter-associated bacteremia (no/yes)	93/7	96/4	.352
PEEP 24 hours after IMV (cmH_2_O)	13 (12–14)	12 (10–12)	<.001
Plateau pressure 24 hours after IMV (≤30 cmH_2_O/>30 cmH_2_O)	63/37	84/16	<.001
Driving pressure 24 hours after IMV (≤15 cmH_2_O/>15 cmH_2_O)	33/67	46/54	.060
IMV LOS (days)	11 (8–19)	7 (4–13)	<.001
ICU LOS (days)	12 (8–18)	8 (4–12.25)	<.001
Hospital LOS (days)	21 (16–28)	18 (13–27.50)	.152
In-hospital mortality (survive/death)	61/39	88/12	<.001

Date are presented as n or median (IQR).

ICU = intensive care unit, IMV = invasive mechanical ventilation, LOS = length of stay, P/FP = PaO_2_/(FiO_2_*PEEP).

Data on the immunosuppression, sequential organ failure assessment, and arrhythmia were missing for 1 patient, on the C-reactive protein for 10 patients, on the procalcitonin for 64 patients, on the creatinine phosphokinase-total for 18 patients, on the creatinine phosphokinase-MB for 12 patients.

### 3.2. Univariate analysis of in-hospital mortality and sensitivity analyses

In the univariate analysis, 12 factors were associated with an increased risk of in-hospital mortality, including diabetes, chronic kidney disease, heart failure, immunosuppression, lung damage on computed tomography >50%, sequential organ failure assessment score >4, tocilizumab treatment, renal replacement therapy, septic shock, acute kidney failure, pneumonia associated with IMV, and plateau pressure 24 hours after IMV (Table S1, Supplemental Digital Content, http://links.lww.com/MD/N663). Those with a univariate *P*-value of .05 and other clinically relevant factors were included in the multivariate analysis.

We also performed a stratified analysis based on baseline characteristics. As shown in Table S2, Supplemental Digital Content, http://links.lww.com/MD/N663 in subgroup analyses, the high P/FP group had a lower in-hospital mortality rate in most strata compared with the low P/FP group. We did not conduct stratified analysis when the sample size was <15. The interaction test was statistically significant (*P* < .001) for both the lymphocyte group and the acute kidney failure group (Table S2, Supplemental Digital Content, http://links.lww.com/MD/N663 and Table S3, Supplemental Digital Content, http://links.lww.com/MD/N663). Patients in the lymphocytes ≤1 × 10^9^/L group and the acute kidney failure group had a higher risk of death.

### 3.3. Multivariate analyses of P/FP and in-hospital mortality

**Figure 1.**
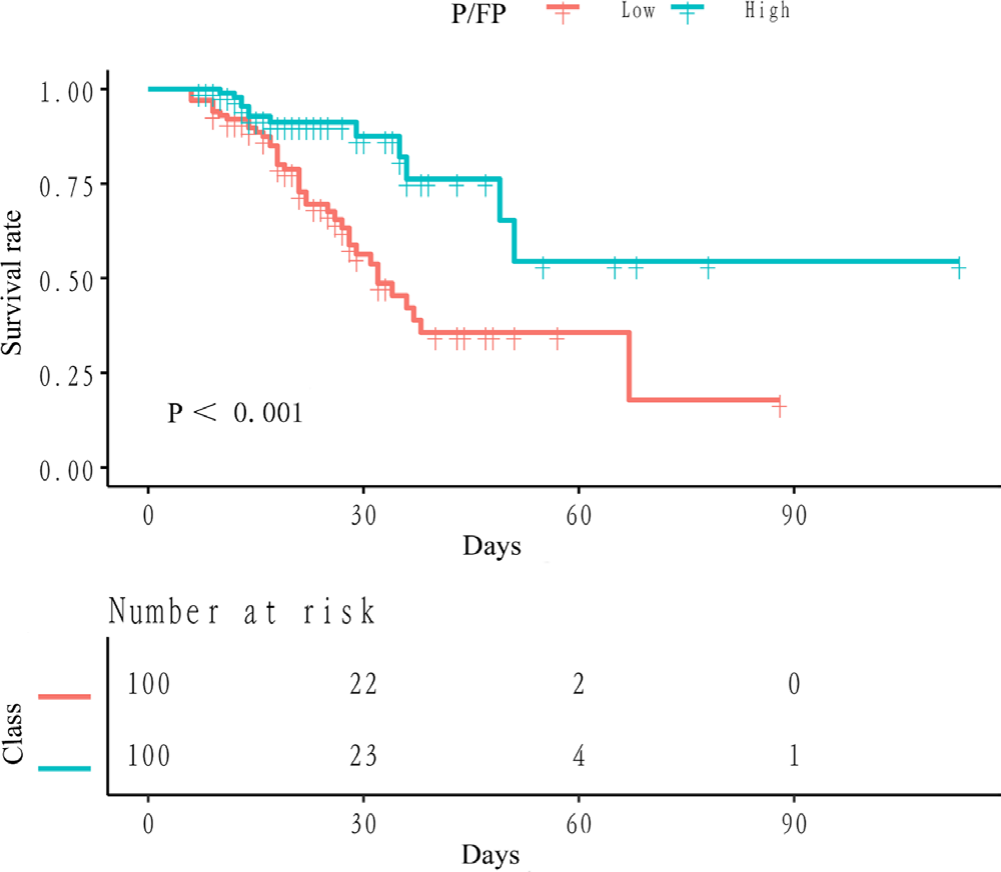
Kaplan–Meier curves for patients in different PaO_2_/(FiO_2_*PEEP) groups.

Of the 200 patients, 51 (25.50%) died in hospital. The Kaplan–Meier curves (Fig. 1) showed a significantly higher cumulative mortality rate in the low P/FP group than in the high P/FP group (*P* < .001). As shown in Table 2, the risk of death was reduced by 67% in the high P/FP group compared with the low P/FP group (unadjusted hazard ratio [HR]: 0.33; 95% confidence interval [CI]: 0.17–0.63). The relationship remained solid after adjusting for confounding factors (HR: 0.10; 95% CI: 0.02–0.47). We also found a significant negative relationship between per P/FP and in-hospital mortality after adjusting for confounding variables (HR: 0.74; 95% CI: 0.65–0.85).

**Table 2:** Cox regression model for in-hospital mortality.

Variable	Unadjusted HR(95% CI)	Adjusted I HR(95% CI)	Adjusted II HR(95% CI)
Initial cohort			
Low (P/FP < 20.50)	1.0	1.0	1.0
High (P/FP ≥ 20.50)	0.33 (0.17, 0.63)	0.17 (0.05, 0.56)	0.10 (0.02, 0.47)
Per P/FP	0.90 (0.86, 0.95)	0.77 (0.69, 0.87)	0.74 (0.65, 0.85)
After MI			
Low (P/FP < 20.50)	1.0	1.0	1.0
High (P/FP ≥ 20.50)	0.33 (0.17, 0.63)	0.41 (0.18, 0.95)	0.35 (0.15, 0.84)
Per P/FP	0.90 (0.86, 0.95)	0.90 (0.83, 0.96)	0.89 (0.83, 0.96)

CI = confidence interval, HR = hazard ratio, IMV = invasive mechanical ventilation, MI = multiple imputation, P/FP = PaO_2_/(FiO_2_*PEEP).

Model I adjust for: age, hypertension, diabetes, chronic kidney disease, heart failure, immunosuppression, C-reactive protein, procalcitonin, alanine aminotransferase, aspartate aminotransferase, lung damage on computed tomography, sequential organ failure assessment, colchicine, tocilizumab, renal replacement therapy, sepsis, septic shock, acute kidney failure, arrhythmia, pneumonia associated with IMV, plateau pressure 24 hours after IMV, driving pressure 24 hours after IMV.

Model II adjust for: gender, age, obesity, hypertension, diabetes, chronic kidney disease, heart failure, asthma, immunosuppression, white blood cells, lymphocytes, C-reactive protein, procalcitonin, alanine aminotransferase, aspartate aminotransferase, creatinine phosphokinase-total, creatinine phosphokinase-MB, lung damage on computed tomography, sequential organ failure assessment, colchicine, tocilizumab, renal replacement therapy, sepsis, septic shock, acute kidney failure, arrhythmia, pneumonia associated with IMV, catheter-associated bacteremia, plateau pressure 24 hours after IMV, driving pressure 24 hours after IMV.

We also performed MI to reduce missing data biases and maximize statistical power. The MI was based on 5 replications and the Markov chain Monte Carlo method in the MI procedure in R to account for missing data for C-reactive protein, procalcitonin, creatinine phosphokinase-total, and creatinine phosphokinase-MB. Results were similar to those of the initial cohort adjusted for potential confounders (Table 2).

### 3.4. Nonlinear relationship and threshold effect of P/FP on in-hospital mortality

**Figure 2.**
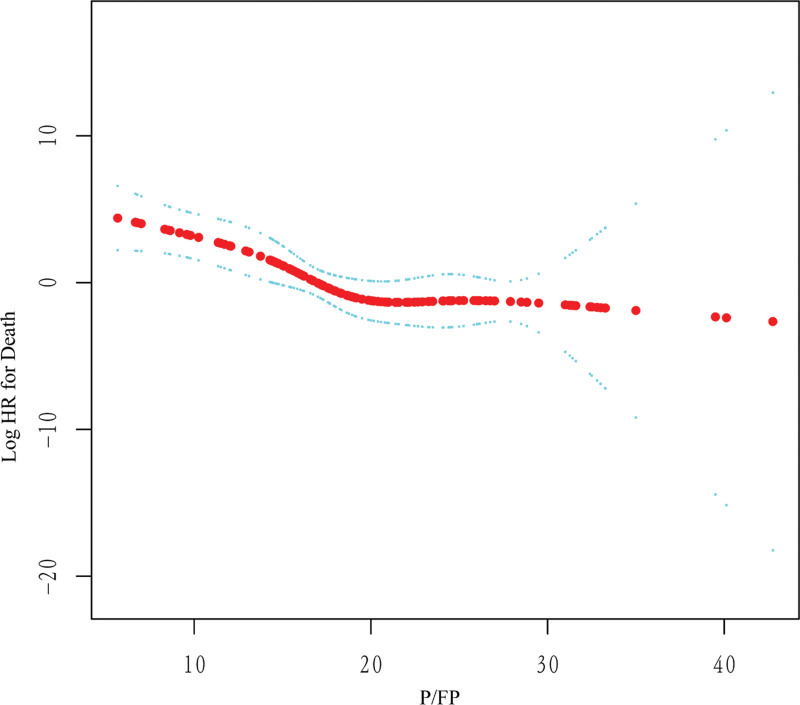
Smoothed curve between PaO_2_/(FiO_2_*PEEP) and in-hospital mortality. Adjusted for gender, age, obesity, hypertension, diabetes, chronic kidney disease, heart failure, asthma, immunosuppression, white blood cells, lymphocytes, C-reactive protein, procalcitonin, alanine aminotransferase, aspartate aminotransferase, creatinine phosphokinase-total, creatinine phosphokinase-MB, lung damage on computed tomography, sequential organ failure assessment, colchicine, tocilizumab, renal replacement therapy, sepsis, septic shock, acute kidney failure, arrhythmia, pneumonia associated with IMV, catheter-associated bacteremia, plateau pressure 24 hours after IMV, driving pressure 24 hours after IMV. IMV = invasive mechanical ventilation.

After adjusting for potential confounders, a nonlinear relationship between P/FP and in-hospital mortality was observed (Fig. 2). Before the turning point (P/FP = 22 mm Hg/cmH_2_O), the risk of death decreased with P/FP. After the turning point ((P/FP = 22 mm Hg/cmH_2_O), the risk of death did not decrease with P/FP. The threshold effect of P/FP on in-hospital mortality was significant with adjustment for the confounders (*P* = .043). The HR was 0.67 (95% CI: 0.56–0.79) for P/FP ≤ 22 mm Hg/cmH_2_O and 1.10 (95% CI: 0.83–1.47) for P/FP > 22 mm Hg/cmH_2_O (Table 3).

**Table 3:** Threshold effect analysis of PaO_2_/(FiO_2_*PEEP) and in-hospital mortality.

Models	Per-unit increase	
	Adjusted HR (95% CI)	*P*-value
Model I		
One line effect	0.74 (0.65, 0.85)	<.001
Model II		
Turning point (K)	22	
Duration of IMV ≤ K	0.67 (0.56, 0.79)	<.001
Duration of IMV > K	1.10 (0.83, 1.47)	.507
*P* value for LRT test	.043	

CI = confidence interval, HR = hazard ratio, IMV = invasive mechanical ventilation.

Adjusted for gender, age, obesity, hypertension, diabetes, chronic kidney disease, heart failure, asthma, immunosuppression, white blood cells, lymphocytes, C-reactive protein, procalcitonin, alanine aminotransferase, aspartate aminotransferase, creatinine phosphokinase-total, creatinine phosphokinase-MB, lung damage on computed tomography, sequential organ failure assessment, colchicine, tocilizumab, renal replacement therapy, sepsis, septic shock, acute kidney failure, pneumonia associated with IMV, catheter-associated bacteremia, plateau pressure 24 hours after IMV, driving pressure 24 hours after IMV.

## 4. Discussion

By reanalyzing published data from Peru, we found that the low P/FP group was associated with a higher risk of death compared to the high P/FP group in patients with ARDS due to COVID-19. Part of the reason for this might be that these patients who required higher PEEP levels to prevent recurrent alveolar collapse might have more severe lung damage.^[10]^ Higher PEEP values could impaired cardiac function, including both systolic and diastolic dysfunction, which might also have contributed to the higher mortality.^[11–14]^ The prone position and high PEEP ventilation strategies might require heavy sedation, which could lead to complications such as ventilation-associated pneumonia, prolonged IMV duration, and ICU-acquired weakness, potentially prolonging ICU and hospital stays and further increasing mortality.^[15,16]^ Unfortunately, due to limitations of the secondary study, data on cardiac insufficiency and the use of sedatives, analgesics, and neuromuscular blocking agents were not available for further analysis.

After adjusting for potential confounders, we found a nonlinear relationship between P/FP after 24 hours of IMV and in-hospital mortality at the inflexion point of 22 mm Hg/cmH_2_O. To the best of our knowledge, this clinical study firstly demonstrated that P/FP after 24 hours of IMV had a nonlinear association with in-hospital mortality in patients with ARDS due to COVID-19.

In addition, patients in the lymphocytes ≤1 × 10^9^/L group and acute kidney failure group had a higher risk of death. Lymphopenia was associated with poor prognosis in COVID-19 patients,^[17,18]^ which was consistent with our study. Kuiper et al^[19]^ proposed that IMV might trigger or exacerbate acute renal failure through 3 mechanisms: firstly, patients requiring IMV were often comorbid with hypoxemia, which impaired renal blood flow; secondly, IMV affected renal hemodynamics by influencing cardiac output; and thirdly, ventilator-associated inflammatory mediators caused the renal injury. Acute renal failure increases the risk of death in patients with IMV.^[20,21]^ In addition, it had been shown that the use of high PEEP in COVID-19 ICU patients was associated with a 5-fold increase in the risk of acute kidney failure, leading to higher mortality.^[22]^ In our study, the low P/FP group had a higher proportion of acute kidney injury and higher PEEP levels, which might be partly responsible for the higher mortality rate in patients in the low P/FP group.

There were still some limitations in this study. First, the data were collected from a single center in Peru. Therefore, our results might not be generalized to other regions. Second, residual bias and unmeasured confounding factors were unavoidable.

## 5. Conclusions

After adjusting for potential confounders, the P/FP after 24 hours of IMV was nonlinearly associated with in-hospital mortality in patients with ARDS due to COVID-19.

## Author contributions

**Investigation:** Jinhuang Lin.

**Methodology:** Huangen Li, Tianlai Lin.

**Project administration:** Zhiwei Su, Tianlai Lin.

**Software:** Youli Chen, Huangen Li, Tianlai Lin.

**Visualization:** Jinhuang Lin.

**Writing – original draft:** Youli Chen.

**Writing – review & editing:** Huangen Li, Jinhuang Lin, Zhiwei Su, Tianlai Lin.

## Supplementary Material


