# Supplementary material for: Association between PaO2/(FiO2*PEEP) ratio and in-hospital mortality in COVID-19 patients: A reanalysis of published data from Peru using PaO2/(FiO2*PEEP) ratio in place of PaO2/FaO2 ratio
Source: Medicine (Baltimore). 2024 Oct 4;103(40):e39931. doi: 10.1097/MD.0000000000039931 (PMC11460852; doi:10.1097/MD.0000000000039931)
Supplement: Supplementary file 1 [file medi-103-e39931-s001.docx]

Supplementary Table 1 Univariate analysis of in-hospital mortality

| **Variable** | **HR (95% CI)** | **P-value** |
| --- | --- | --- |
| Gender (Female/Male) | 0.90 (0.45, 1.81) | 0.770 |
| Age (< 65 years/≥ 65 years) | 1.35 (0.76, 2.41) | 0.311 |
| Obesity (no/yes) | 1.16 (0.66, 2.03) | 0.605 |
| Hypertension (no/yes) | 1.21 (0.68, 2.15) | 0.525 |
| Diabetes (no/yes) | 1.91 (1.04, 3.49) | 0.036 |
| Chronic renal insufficiency (no/yes) | 6.86 (3.06, 15.38) | <0.001 |
| Heart failure (no/yes) | 2.85 (1.32, 6.12) | 0.007 |
| Asthma (no/yes) | 1.27 (0.66, 2.44) | 0.469 |
| Immunosuppression (no/yes) | 3.54 (1.80, 6.96) | <0.001 |
| White blood cells (≤ 10 × 10^9^/L/> 10 × 10^9^/L) | 1.46 (0.83, 2.56) | 0.184 |
| Lymphocytes (≤ 1 × 10^9^/L/> 1 × 10^9^/L) | 1.39 (0.75, 2.59) | 0.296 |
| C-reactive protein (≤ 100 mg/L/> 100 mg/L) | 1.87 (0.95, 3.67) | 0.068 |
| Procalcitonin (≤ 0.5 ng/mL/> 0.5 ng/ml) | 1.26 (0.61, 2.58) | 0.535 |
| Alanine aminotransferase (≤ 40 U/L/> 40 U/L) | 0.63 (0.35, 1.12) | 0.114 |
| Aspartate aminotransferase (≤ 40 U/L/> 40 U/L) | 1.01 (0.56, 1.80) | 0.986 |
| Creatinine phosphokinase-Total (≤ 200 U/L/> 200 U/L) | 1.61 (0.91, 2.84) | 0.104 |
| Creatinine phosphokinase-MB (≤ 25 U/L/> 25 U/L) | 1.11 (0.63, 1.97) | 0.712 |
| Lung damage on computed tomography (≤ 50%/> 50%) | 2.89 (1.23, 6.82) | 0.015 |
| Sequential organ failure assessment (≤ 4/> 4) | 3.20 (1.78, 5.76) | <0.001 |
| Colchicine (no/yes) | 1.33 (0.75, 2.36) | 0.335 |
| Tocilizumab (no/yes) | 2.53 (1.31, 4.91) | 0.006 |
| Renal replacement therapy (no/yes) | 3.75 (1.98, 7.14) | <0.001 |
| Sepsis (no/yes) | 1.55 (0.69, 3.47) | 0.286 |
| Septic shock (no/yes) | 2.91 (1.60, 5.29) | <0.001 |
| Acute kidney failure (no/yes) | 3.25 (1.85, 5.71) | <0.001 |
| Arrhythmia (no/yes) | 1.79 (0.85, 3.75) | 0.122 |
| Pneumonia associated with IMV (no/yes) | 3.89 (2.21, 6.84) | <0.001 |
| Catheter-associated bacteremia (no/yes) | 1.40 (0.55, 3.52) | 0.481 |
| Plateau pressure 24 h after IMV (≤ 30 cmH_2_O/> 30 cmH_2_O) | 2.23 (1.28, 3.88) | 0.004 |
| Driving pressure 24 h after IMV (≤ 15 cmH_2_O/> 15 cmH_2_O) | 1.46 (0.81, 2.64) | 0.210 |

HR = hazard ratio, CI = confidence interval, IMV = invasive mechanical ventilation

Supplementary Table 2 Stratified analysis for unadjusted hazard ratio in the subgroups

| **Variable** | **HR (95% CI)** | | **P for interaction** |
| --- | --- | --- | --- |
|  | **Low (P/FP < 20.50)** | **High (P/FP ≥ 20.50)** |  |
| Gender |  |  | 0.162 |
| Female | 1.0 | 0.69 (0.19, 2.47) |  |
| Male | 1.0 | 0.25 (0.11, 0.54) |  |
| Age (years) |  |  | 0.717 |
| < 65 | 1.0 | 0.32 (0.14, 0.71) |  |
| ≥ 65 | 1.0 | 0.41 (0.13, 1.26) |  |
| Obesity |  |  | 0.641 |
| No | 1.0 | 0.26 (0.09, 0.73) |  |
| Yes | 1.0 | 0.37 (0.16, 0.87) |  |
| Hypertension |  |  | 0.188 |
| No | 1.0 | 0.44 (0.21, 0.92) |  |
| Yes | 1.0 | 0.17 (0.04, 0.73) |  |
| Diabetes |  |  | 0.236 |
| No | 1.0 | 0.39 (0.18, 0.85) |  |
| Yes | 1.0 | 0.18 (0.05, 0.66) |  |
| Chronic renal insufficiency |  |  | - |
| No | 1.0 | 0.38 (0.20, 0.75) |  |
| Yes | - | - |  |
| Heart failure |  |  | - |
| No | 1.0 | 0.36 (0.19, 0.71) |  |
| Yes | - | - |  |
| Asthma |  |  | 0.613 |
| No | 1.0 | 0.35 (0.17, 0.73) |  |
| Yes | 1.0 | 0.26 (0.06, 1.19) |  |
| Immunosuppression |  |  | - |
| No | 1.0 | 0.39 (0.20, 0.79) |  |
| Yes | 1.0 | 0.21 (0.03, 1.64) |  |
| White blood cells (× 10^9^/L) |  |  | 0.036 |
| ≤ 10 | 1.0 | 0.59 (0.25, 1.42) |  |
| > 10 | 1.0 | 0.16 (0.06, 0.47) |  |
| Lymphocytes (× 10^9^/L) |  |  | 0.015 |
| ≤ 1 | 1.0 | 0.50 (0.24, 1.01) |  |
| > 1 | 1.0 | 0.08 (0.01, 0.57) |  |
| C-reactive protein (mg/L) |  |  | 0.054 |
| ≤ 100 | 1.0 | 0.83 (0.25, 2.74) |  |
| > 100 | 1.0 | 0.18 (0.06, 0.52) |  |
| Procalcitonin (ng/ml) |  |  | 0.996 |
| ≤ 0.5 | 1.0 | 0.15 (0.05, 0.42) |  |
| > 0.5 | 1.0 | 0.25 (0.03, 1.97) |  |
| Alanine aminotransferase (U/L) |  |  | 0.586 |
| ≤ 40 | 1.0 | 0.42 (0.13, 1.28) |  |
| > 40 | 1.0 | 0.28 (0.12, 0.65) |  |
| Aspartate aminotransferase (U/L) |  |  | 0.583 |
| ≤ 40 | 1.0 | 0.24 (0.08, 0.75) |  |
| > 40 | 1.0 | 0.37 (0.17, 0.83) |  |
| Creatinine phosphokinase-Total (U/L) |  |  | 0.545 |
| ≤ 200 | 1.0 | 0.35 (0.15, 0.82) |  |
| > 200 | 1.0 | 0.22 (0.06, 0.75) |  |
| Creatinine phosphokinase-MB (U/L) |  |  | 0.865 |
| ≤ 25 | 1.0 | 0.26 (0.09, 0.71) |  |
| > 25 | 1.0 | 0.32 (0.12, 0.84) |  |
| Lung damage on computed tomography |  |  | 0.129 |
| ≤ 50% | 1.0 | 1.25 (0.23, 6.85) |  |
| > 50% | 1.0 | 0.29 (0.13, 0.62) |  |
| Sequential organ failure assessment |  |  | 0.089 |
| ≤ 4 | 1.0 | 0.62 (0.23, 1.62) |  |
| > 4 | 1.0 | 0.24 (0.09, 0.69) |  |
| Colchicine |  |  | 0.739 |
| No | 1.0 | 0.34 (0.16, 0.72) |  |
| Yes | 1.0 | 0.32 (0.07, 1.39) |  |
| Tocilizumab |  |  | 0.580 |
| No | 1.0 | 0.33 (0.16, 0.68) |  |
| Yes | 1.0 | 0.80 (0.15, 4.23) |  |
| Renal replacement therapy |  |  | - |
| No | 1.0 | 0.49 (0.25, 0.97) |  |
| Yes | - | - |  |
| Sepsis |  |  | 0.398 |
| No | 1.0 | 0.57 (0.11, 2.85) |  |
| Yes | 1.0 | 0.29 (0.13, 0.62) |  |
| Septic shock |  |  | 0.376 |
| No | 1.0 | 0.44 (0.16, 1.21) |  |
| Yes | 1.0 | 0.31 (0.12, 0.80) |  |
| Acute kidney failure |  |  | 0.008 |
| No | 1.0 | 0.58 (0.28, 1.20) |  |
| Yes | 1.0 | 0 (0, Inf) |  |
| Arrhythmia |  |  | - |
| No | 1.0 | 0.28 (0.13, 0.59) |  |
| Yes | - | - |  |
| Pneumonia associated with IMV |  |  | 0.815 |
| No | 1.0 | 0.36 (0.13, 0.98) |  |
| Yes | 1.0 | 0.34 (0.14, 0.83) |  |
| Catheter-associated bacteremia |  |  | - |
| No | 1.0 | 0.33 (0.17, 0.65) |  |
| Yes | - | - |  |
| Plateau pressure 24 h after IMV (cmH_2_O) |  |  | 0.498 |
| ≤ 30 | 1.0 | 0.31 (0.13, 0.72) |  |
| > 30 | 1.0 | 0.50 (0.17, 1.47) |  |
| Driving pressure 24 h after IMV (cmH_2_O) |  |  | 0.299 |
| ≤ 15 | 1.0 | 0.12 (0.03, 0.54) |  |
| > 15 | 1.0 | 0.43 (0.19, 0.94) |  |

HR = hazard ratio, CI = confidence interval, P/FP = PaO_2_/(FiO_2_*PEEP), IMV = invasive mechanical ventilation

Supplementary Table 3 Stratified analysis between PaO_2_/(FiO_2_*PEEP) and in-hospital mortality

| **Exposure** | **Variable** | **Unadjusted** **HR(95% CI)** | **Adjusted HR(95% CI)** |
| --- | --- | --- | --- |
|  | Lymphocytes (× 10^9^/L) |  |  |
| N |  | 200 | 125 |
| Low P/FP group | ≤ 1 | 1.0 | 1.0 |
| High P/FP group | ≤ 1 | 0.50 (0.24, 1.01) | 0.42 (0.15, 1.17) |
| Low P/FP group | > 1 | 2.31 (1.18, 4.54) | 0.78 (0.36, 1.66) |
| High P/FP group | > 1 | 0.13 (0.02, 0.96) | 0 (0, Inf) |
| P for interaction |  | 0.015 | <0.001 |
|  | White blood cells (× 10^9^/L) |  |  |
| N |  | 200 | 125 |
| Low P/FP group | ≤ 10 | 1.0 | 1.0 |
| High P/FP group | ≤ 10 | 0.65 (0.27, 1.56) | 0.17 (0.02, 1.56) |
| Low P/FP group | > 10 | 2.10 (1.09, 4.05) | 1.14 (0.26, 4.98) |
| High P/FP group | > 10 | 0.33 (0.11, 1.01) | 0.06 (0, 0.69) |
| P for interaction |  | 0.036 | 0.475 |
|  | Acute kidney failure |  |  |
| N |  |  |  |
| Low P/FP group | No | 1.0 | 1.0 |
| High P/FP group | No | 0.59 (0.28, 1.23) | 0.67 (0.25, 1.85) |
| Low P/FP group | Yes | 3.41 (1.81, 6.42) | 1.20 (0.62, 2.33) |
| High P/FP group | Yes | 0 (0, Inf) | 0 (0, Inf) |
| P for interaction |  | 0.008 | < 0.001 |

HR = hazard ratio, CI = confidence interval, P/FP = PaO_2_/(FiO_2_*PEEP)

Adjusted for gender, age, obesity, hypertension, diabetes, chronic kidney disease, heart failure, asthma, immunosuppression, white blood cells, lymphocytes, C-reactive protein, procalcitonin, alanine aminotransferase, aspartate aminotransferase, creatinine phosphokinase-Total, creatinine phosphokinase-MB, lung damage on computed tomography, sequential organ failure assessment, colchicine, tocilizumab, renal replacement therapy, sepsis, septic shock, acute kidney failure, pneumonia associated with IMV, catheter-associated bacteremia, plateau pressure 24 h after IMV, driving pressure 24 h after IMV.
